# Advanced Solid Geopolymer Formulations for Refractory Applications

**DOI:** 10.3390/ma17061386

**Published:** 2024-03-18

**Authors:** Shaik Hussain, Sudhir Amritphale, John Matthews, Niloy Paul, Elizabeth Matthews, Richard Edwards

**Affiliations:** 1 Trenchless Technology Center, Louisiana Tech University, Ruston, LA 71272, USA; amritphale57@gmail.com (S.A.); npa024@latech.edu (N.P.); rre014@latech.edu (R.E.); 2 Civil Engineering & Construction Engineering Technology, Louisiana Tech University, Ruston, LA 71272, USA; ematt@office.latech.edu

**Keywords:** advanced solid geopolymer, refractory, thermal fatigue resistance, compressive strength

## Abstract

Cement, as a construction material, has low thermal resistance, inherent fire resistance, and is incombustible up to a certain degree. However, the loss of its mechanical performance and spalling are its primary issues, and it thus cannot retain its performance in refractory applications. The present study explores the performance of geopolymer formulations that have excellent fire resistance properties for potential refractory applications. This study is unique, as it investigates advanced solid geopolymer formulations that need only water to activate and bind. Various solid geopolymer formulations with fly ash as a precursor; potassium hydroxide and potassium silicate as activators; and mullite and alumina as refractory aggregates were studied for their compressive strength at up to 1100 °C and compared with their two-part conventional liquid alkaline geopolymer counterparts. Advanced solid geopolymer formulations with mullite and alumina as refractory aggregates had mechanical strength values of 84 MPa and 64 MPa post-1100 °C exposure and were further exposed to ten thermal cycles of 1100 °C to study their fatigue resistance and post-exposure compressive strengths. The geopolymer sample with mullite as a refractory aggregate yielded 115.2 MPa compressive strength after the fourth cycle of exposure. This sample was also studied for its temperature distribution upon direct flame exposure. All the geopolymer formulations displayed a drop in compressive strength at 600 °C due to viscous sintering and then a rise in strength at 1100 °C due to phase transformation. X-ray diffraction studies revealed that the formation of crystalline phases such as leucite, sanidine, and annite were responsible for the superior strengths at 1100 °C for the alumina- and mullite-based geopolymer formulations.

## 1. Introduction

Portland cement and its products, such as cement mortar or concrete, do not achieve the desired performance when subjected to elevated temperatures. Damage in these structures is usually observed in the form of cracks typically induced by pore pressure build-up and thermal stresses [1,2,3]. Therefore, the performance of cement concrete constructions under the influence of fire and high temperatures has been extensively studied to mitigate thermally induced damage [4,5,6,7,8,9]. Portland cement and its hydration products, such as calcium hydroxide, calcium silicate hydrates, and calcium carbonate, degrade at various temperatures, making it impossible for the microstructure of cement products to retain thermal stability. At 100 °C, physically evaporable water from the pore structure is lost, followed by a risk of spalling at a temperature range from 200 °C to 400 °C. The aggregates used in cement concrete lose their strength after reaching a temperature of 300 °C. The hydration products calcium hydroxide and calcium silicate hydrates undergo disassociation at 450 °C and 600 °C, respectively. Calcium carbonate decarbonates at a temperature range of 700 °C to 800 °C [10]. At a temperature of 800 °C, all chemically bound water is completely evaporated too [11]. Hence, there is a dearth of construction material that can sustain and retain its strength after a temperature exposure of 800 °C.

Geopolymers are a sustainable and green alternative to Portland cement that is produced by activating industrial wastes or by-products such as fly ash, slag, red mud, etc. Apart from being a value-added product from industrial waste/by-products, geopolymers also mitigate the high carbon dioxide release that is usually prominent in cement manufacturing [12,13]. Also, compared with Portland cement concrete, geopolymer products display superior and sustained structural integrity at elevated temperatures [14]. 

The chemistry of geopolymerization is closely related to alkali activation. Initial theories of alkali activation included several stages, such as destruction, coagulation, condensation, and crystallization [15]. Further researchers [16,17] have postulated dissolution, gelation, reorganization, and polymerization as major steps in geopolymerization. Si-O-Si, Al-O-Al, and Al-O-Si bonds in aluminosilicate-rich materials are broken in the presence of a strong alkaline medium, generating alkaline silicates that cause dissolution. Alkaline silicates may form Si-O-Ca-OH bonds when they interact with divalent ions. Al(OH)_4_^−^, Al(OH)_5_^2−^, and Al(OH)_6_^3−^ are formed depending on the pH value of the Al-O-Si bonds. These products are de-segregated and eventually come closer to polycondensation. After geopolymerization, in the final stage, a synthetic three-dimensional alkali aluminosilicate is formed. Geopolymers are usually made from aluminosilicate sources that are abundantly available in industrial by-products such as fly ash, metakaolin, blast furnace slag, red mud, etc., which are activated by alkaline hydroxides and silicates of sodium or potassium. Precursors in geopolymers can be classified based on their calcium oxide content; Class F fly ash and metakaolin fall under the category of low-calcium-oxide-based precursors, whose main reaction product is a three-dimensional inorganic alkaline polymer based on sodium aluminosilicate hydrate (N-A-S-H) gel. Blast furnace slag-based precursors form a gel similar to the hydration product C-S-H, calcium aluminosilicate hydrate (C-A-S-H), with a small percentage of Al in it. Compared with calcium silicate hydrates and calcium hydroxide from Portland cement products, non-hydrated alumino-silicate binding gels in geopolymers do not contain as much physically or chemically bound water; hence, higher resistance to temperatures of up to 1200 °C are achieved in geopolymers due to their open-pore structures [18,19]. 

At higher temperatures, both cement concrete and geopolymer concrete are subjected to similar conditions that result in three different mechanisms. The failure in the concrete can either be due to thermal incompatibility, phase transformation, or pore pressure buildup [10,20,21,22]. Thermal incompatibility is the result of a varying degree of expansion and contraction of the aggregates in concrete and cement or the geopolymer matrix. Aggregates expand, and the matrix shrinks at an elevated temperature range, leading to a debonding in the interfacial transition zone and crack initiation [23,24]. Phase transformations at elevated temperatures are due to the removal of physically and chemically bound water at various temperature ranges, dehydroxylation, and decarbonation. Also, calcium silicate hydrates decompose at a temperature of 600 °C [10,11]. Pore pressure buildup is another significant reason for crack initiation and failure in concrete. Up until 100 °C, the concrete specimen will have moisture in its pores. As the temperature rises, the moisture flows from the hot regions of the concrete to the colder regions, leading to the accumulation of moisture. Since the inner regions of concrete have lower temperatures, the accumulated moisture condenses and forms a moisture clog [2,25,26]. As the accumulated water vapor expands, a significant amount of potential energy is generated in the pores, leading to pore pressure buildup. Eventually, when the pore pressure is sufficiently high, it exceeds the maximum permissible stresses in the concrete, leading to crack initiation or the violent release of energy. Also, irregular and non-uniform compression and tension in the layers of concrete due to varying temperatures can lead to failure [27,28].

Geopolymer concrete displays better temperature resistance and performance compared with cement concrete [18,29]. This is because cement concrete hydration products are based on the supply of water for hydration, which eventually evaporates at higher temperatures, and the geopolymer products are aluminosilicate gels that are non-hydrated in nature and do not hold physically or chemically bound water like calcium silicate hydrate gels [18]. Therefore, an open pore structure in geopolymer products leads to better resistance to temperature for up to 1200 °C with comparably less loss in strength [19]. Aggregates play a predominant role in the heat resistance capacity of concrete and mortar from both cement and geopolymer. Carbonate-based aggregates have better heat absorption capacity compared with siliceous-based aggregates after an exposure temperature of 600 °C [30]. Aggregates such as expanded clay have also been reported to have successfully mitigated thermal incompatibility. 

The present study deals with the performance of refractory geopolymers with from mullite and alumina as aggregates. Class F fly ash was used as a precursor in the geopolymer formulations adopted in this study because of its efficient resistance to vapor pressure buildup due to its porous structure. Metakaolin and slag were not used as precursors in the present study given their denser structure and the decarbonation of calcite, respectively. The novelty of this study lies in the activator used and the method of producing geopolymers. Most studies [24,31,32,33,34,35,36,37,38,39] on geopolymer samples exposed to higher temperatures deal with sodium hydroxide and sodium silicate as activators. Although the sodium sources for alkaline are preferable for regular geopolymer applications, such as construction purposes, they fail to retain their amorphous nature at elevated temperature ranges. Hence, potassium hydroxide and potassium silicate were used as activators in the present study due to their ability to retain their amorphous nature. As the handling of liquid alkaline activators is risky and dangerous and the preparation is exothermic in nature, the present study adopts a unique novel procedure of mechano-chemically grinding the precursors and activators together sequentially in a pre-determined order to make an advanced solid geopolymer powder that needs only water to activate the polymerization reaction. The solid geopolymer powder is easy to mix and has minimum mixing variability, thereby providing consistency to the mix. The simplicity of the solid geopolymer mix reduces time and saves labor costs, making the process economical. The transportability of the solid geopolymer compared with the two-part conventional geopolymer that includes precursors and liquid activators is less cumbersome. The results for advanced solid geopolymer systems are further compared with their two-part conventional liquid alkaline geopolymer counterparts. A few design mix proportions adopted in this study also used rice husk silica, a potential precursor, in place of potassium silicate to explore the possibility of a single activator system. 

## 2. Experimental Details

### 2.1. Raw Material Properties

Fly ash was chosen as a primary aluminosilicate source for the geopolymer mix. Low-calcium-oxide-based fly ash of Class F conforming to ASTM C618-08 [40] was used in the present study. The fly ash was purchased from Salt River Materials Group, Arizona, United States. The chemical composition of fly ash from X-ray fluorescence analysis and loss on ignition and its physical characteristics are presented in Table 1. More than 95% of the fly ash particles passed through a 45-micron sieve.

Mullite (3Al_2_O_3_·2SiO_2_) and alumina (Al_2_O_3_) were used as aggregates for the geopolymer mixes due to their refractory natures. These aggregates were purchased from Noah Chemicals, Texas, United States. The chemical composition of the refractory aggregates used in this study from X-ray fluorescence analysis is tabulated in Table 2. Mullite aggregates of two sizes, 45 microns and 150 microns, and alumina aggregate passed through a 1.18 mm sieve were used in this study. 

Due to the inability of sodium activators to retain an amorphous nature at higher temperatures, potassium activators were used in this study. Commercially available potassium hydroxide flakes bought from Albemarle International Corporation, Baton Rouge, United States, and potassium silicate beads purchased from PQ Corporation, Pennsylvania, United States, in dry form and, in some mixes, liquid form mixed with water were used as activators. The modulus ratio (SiO_2_/K_2_O) of potassium silicate (K_2_SiO_3_) used in the study was 1.606 with SiO_2_ at 51.42% and K_2_O at 32%. The potassium hydroxide (KOH) had a purity of more than 90%. The ratios of potassium silicate and potassium hydroxide in dry and liquid mixes were different.

### 2.2. Design Mix Proportions

Two methodologies of design mix proportions were adopted in this study: a conventional two-part liquid-alkaline-based geopolymer formulation and an advanced solid geopolymer powder formulation. 

For the conventional two-part liquid-alkaline-based geopolymer formulation, dry potassium hydroxide and potassium silicate were mixed with water to prepare the solution 24 h in advance. Then, a 50% potassium silicate solution was prepared using 50% distilled water and 50% solid potassium silicate. The potassium hydroxide solution used in this study had a molarity of 12.5 M with 700 g/L (12.5 × 56), where 56 is the molecular weight of KOH. The design mix proportions of the conventional two-part liquid alkaline geopolymer formulations are tabulated in Table 3 with the mix names LR1, LR2, and LR3.

**Figure 1:**
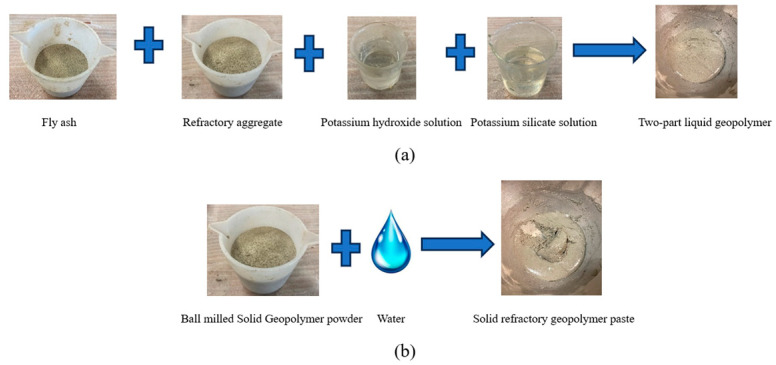
Pictorial representation of (**a**) a conventional two-part geopolymer and (**b**) a solid refractory geopolymer.

For advanced solid geopolymer formulation, the precursor, Class F fly ash, and the activators, dry potassium hydroxide and potassium silicate, were mechano-chemically ground in a ball mill for a pre-determined time ranging from 15 min to 5 h depending on the rpm of the mixer or ball mill in a sequential order of activators and then activators together with precursors. The solid dry powder, which was submicron- to nano-sized, needed only water to activate the geopolymerization reaction. Figure 1 shows a pictorial representation of a conventional two-part geopolymer process and advanced solid geopolymer formulation. 

The solid geopolymer powder mixes are denoted as SR1, SR2, SR3, and SR4 in Table 3. In the design mix, denoted as SR4, rice husk silica (RHS) was used along with potassium hydroxide as an activator. This mix explores the possibility of obtaining potassium silicate from a reaction between rice husk silica and the extra potassium hydroxide used in the mix. Also, mixes denoted by LR1 and SR1 had a mullite aggregate with a particle size of 45 microns, and LR2, SR2, and SR4 had a mullite aggregate with a particle size of 150 microns. Mixes denoted by LR3 and SR3 used dense alumina passed through a 1.18 mm sieve as an aggregate.

All the geopolymer samples were cured at 70 °C for 24 h before curing them at room temperature for 6 more days, completing a curing period of 7 days before exposing them to elevated temperatures.

### 2.3. Experimental Methodology

The heating rate and cooling rate conditions have prominent effects on the performance of refractory geopolymer composites. Hardened geopolymer samples can be subjected to a constant heating rate or a simulation of a real fire situation. Real fire situations lead to a rapid and unstable increase in temperature in a short duration of time and can be responsible for an erratic temperature gradient. A constant heating rate, however, can minimize the thermal stress and gradient. Heating rates between 1 °C/min to 10 °C/min have been adopted in literature [25,41,42]. 

**Figure 2:**
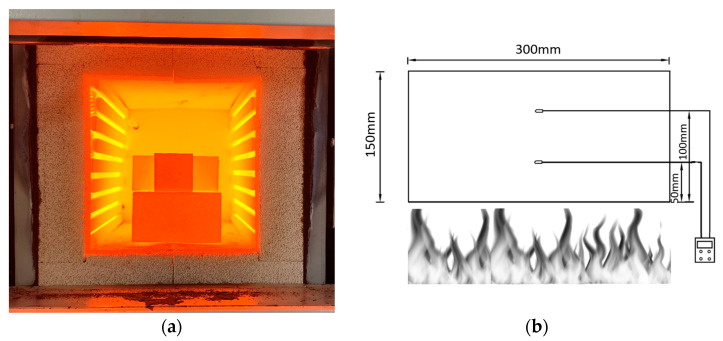
(**a**). SR1 specimen exposed to 1100 °C. (**b**) Temperature distribution by direct flame.

In the present study, a constant heating rate of 5 °C/min in a furnace was adopted, and after the specimens reached the desired temperature, they were maintained at that temperature for 60 min and then gradually allowed to cool down naturally, as per ambient conditions. Sudden or rapid cooling and cooling by water has been reported to decrease strength by more than 15% when exposed to 1100 °C [43]. The geopolymer samples were exposed to 200 °C, 400 °C, 600 °C, 800 °C, and 1100 °C, and their strengths were tested under compression using 2-inch cubes, as per provisions in ASTM C109 [44]. Figure 2a shows the specimen of mix SR1 exposed to 1100 °C in a furnace. 

To measure the temperature distribution of the geopolymer composites, geopolymer samples prepared from mixes LR1 and LR3 were subjected to a real fire situation using flames with a temperature of 800 °C for 30 min. A hardened geopolymer sample of 300 mm in length, 300 mm in breadth, and 150 mm in depth, with two thermocouples embedded at a depth of 50 mm from the top and 50 mm from the bottom, was adopted to measure the temperature distribution in relation to time. Figure 2b shows a schematic representation of the sample exposed to flame and the position of thermocouples. 

## 3. Results and Discussion

### 3.1. Physical Appearance and Weight Loss

**Figure 3:**
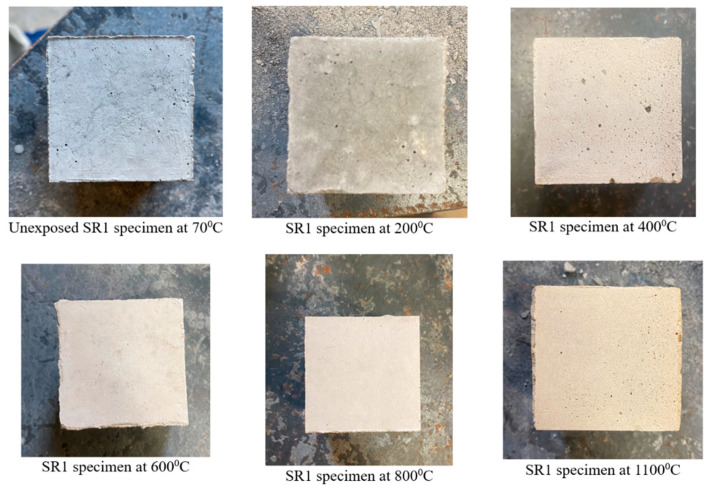
Specimens of mix SR1 before and after temperature exposure.

**Figure 4:**
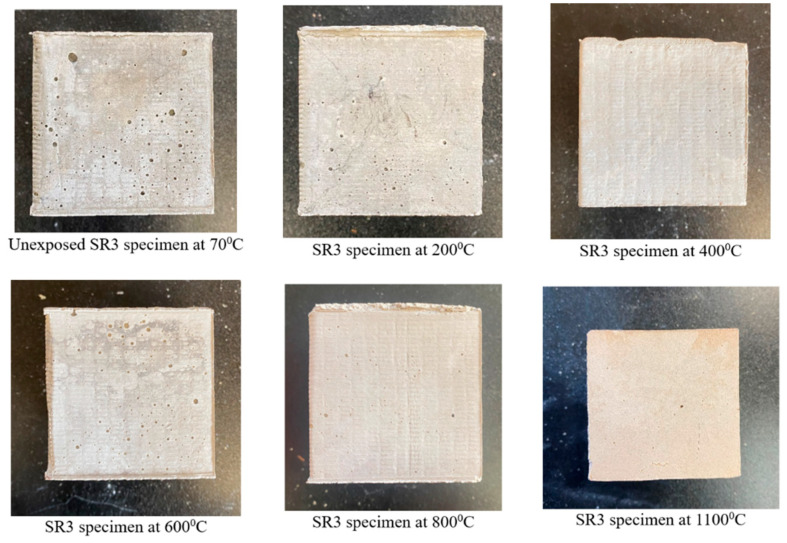
Specimens of mix SR3 before and after temperature exposure.

Figure 3 and Figure 4 show the visual appearance of specimens from mixes SR1 and SR3 prior to and post-exposure to elevated temperatures, respectively. The mechanical performance at high-temperature exposures of mixes SR1 and SR3 was exceptionally better compared with all other mixes that are presented in the preceding sections. Hence, these mixes were chosen for further study regarding thermal cycles to show their visual appearances post-high-temperature exposure and for microstructural analysis. There was no physical deterioration, spalling, or disintegration in the specimens. All specimens retained their cubical shapes post-exposure to high temperatures without any cracking. 

The specimens from mix SR1 displayed changes in color from dark gray at 70 °C to light gray at 200 °C and subsequently to reddish gray as the temperature increased beyond 400 °C. A similar color change was observed in specimens from mix SR3; however, the reddish-gray change started at temperatures beyond 600 °C. The reddish-gray color of the samples exposed to high temperatures is due to the oxidation of iron compounds [45,46,47]. The fly ash used in the present study had iron in the form of iron oxide at 4.73%. 

**Figure 5:**
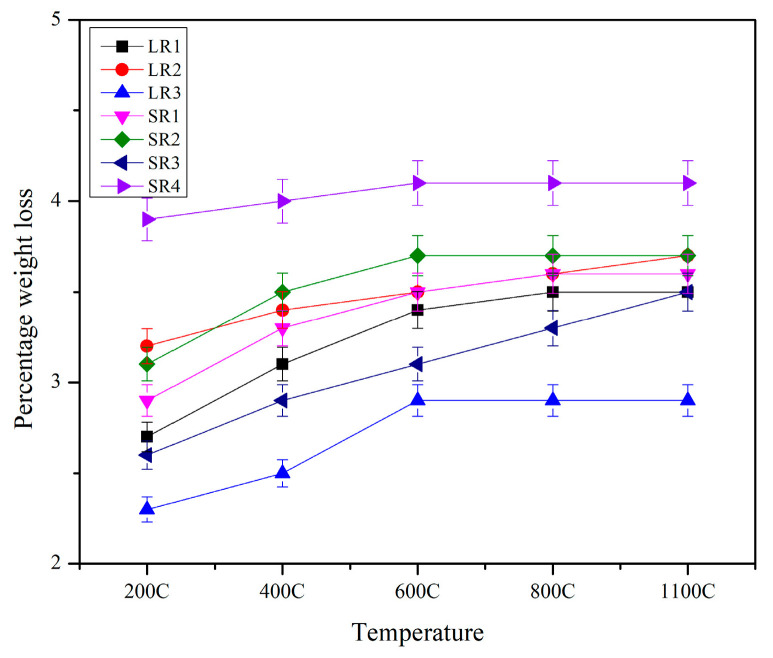
Percentage weight loss of samples exposed to elevated temperatures.

Figure 5 shows the trend of change in weight pre- and post-exposure to elevated temperatures. The percentage loss of weight in the geopolymer samples is due to the evaporation of physically and chemically bound water in alkali and phase transformations. Samples that were prepared using a two-part conventional geopolymerization process (LR1, LR2, and LR3) had less weight loss compared with their advanced solid geopolymer formulation counterparts (SR1, SR2, and SR3). In the latter samples, the geopolymers were prepared by adding water to the powdered mix, unlike the former samples, where the liquid alkaline solutions were prepared 24 h in advance. This resulted in more physically bound available water in the advanced solid geopolymer formulation when compared with the conventional two-part liquid geopolymer, where water was bound chemically. Also, geopolymer samples that had alumina (LR3 and SR3) in them as a refractory aggregate displayed less weight loss compared with the mullite refractory aggregate samples (LR1, LR2, SR1, and SR2). The mullite mixes had a significant amount of alumina and silica in them, resulting in additional geopolymerization due to forming gels at elevated temperatures and thereby leading to shrinkage and more weight loss in these samples compared with alumina mixes that only had abundant amounts of alumina. The geopolymer with 45-micron mullite had denser matrix packing compared with the 150-micron mullite and, hence, had slightly less weight loss. 

### 3.2. Compressive Strength

**Figure 6:**
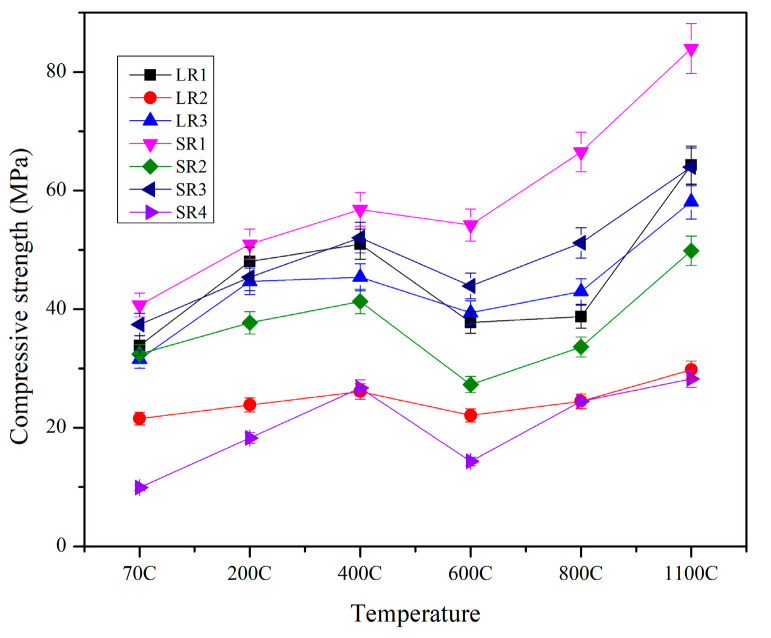
Trend of compressive strength of samples exposed to elevated temperatures.

The plot of compressive strength of the geopolymer samples exposed to elevated temperatures is shown in Figure 6. All the mixes that contained mullite and alumina as refractory aggregates displayed a drop in strength at 600 °C, followed by an increase in strength after 800 °C. The change in compressive strength in the samples exposed to high temperatures can be explained by three phenomena. 

At the initial temperature range of 200 °C to 400 °C, there is a release of pore pressure without any potential cracking due to the porous nature of fly ash particles [47]. This enables further geopolymerization between activators and unreacted fly ash particles forming gels and filling up the cracks, leading to an increase in mechanical strength [47,48]. At 600 °C, the geopolymer matrix undergoes shrinkage and viscous sintering, leading to some fluidity in the geopolymer matrix [49]. This resulted in a decrease in compressive strength. At 800 °C and above, after viscous sintering, micro-cracks heal, leading to matrix densification. At this temperature, there are new stable crystalline phases formed in mixes with both mullite and alumina refractory aggregates [18,50,51]. The mixes that contained mullite as a refractory aggregate saw the formation of sanidine and annite at a temperature of 1100 °C along with cristobalite, which is a high-temperature form of quartz. The mixes that contained alumina as a refractory aggregate showed leucite peaks at 1100 °C. Similar observations were made by Wattanasiriwech et al. [52], who, in their study, concluded that, in a mix with fly ash and mullite, mullite recrystallizes at higher temperatures and forms Si-rich needle-like structures, resulting in greater strength. In a separate study conducted by Ascensao et al. [53], the authors observed a similar trend of decline in strength at 750 °C and a recovery as the temperature further increased. The strength reached a maximum value of 184 MPa at 1100 °C. The authors concluded that CaO-FeO_x_-Al_2_O_3_-SiO_2_-rich phases were responsible for this behavior. Zhang et al. [26] made similar observations, where residual compressive strength decreased after 600 °C for fly ash geopolymer concrete exposed to high temperatures. 

The advanced solid geopolymer mixes displayed better mechanical performance pre- and post-high-temperature exposure when compared with their conventional two-part liquid geopolymer counterparts. The mechano-chemical grinding of the solid powder with precursors and activators together enabled the geopolymer mix to outperform the conventional two-part liquid alkaline geopolymer mix. The moisture loss and, therefore, matrix densification were more pronounced in the advanced solid mix where water was added compared with the two-part mix where a liquid alkaline activator was added. The geopolymer mix denoted as SR1, made of 45-micron mullite, displayed a compressive strength of 84 Mpa post-exposure to 1100 °C. A similar trend was observed for mix SR3, where the sample achieved its highest compressive strength, 64 Mpa, at 1100 °C. Although the trend of compressive strength increasing with temperature reversed at 600 °C, the value of strength was still greater than compressive strength obtained prior to high-temperature exposure for all the mixes. Similar observations were made by Kong et al. [14], who reported that residual compressive strength after elevated-temperature exposure for one hour increased by 6%. 

**Figure 7:**
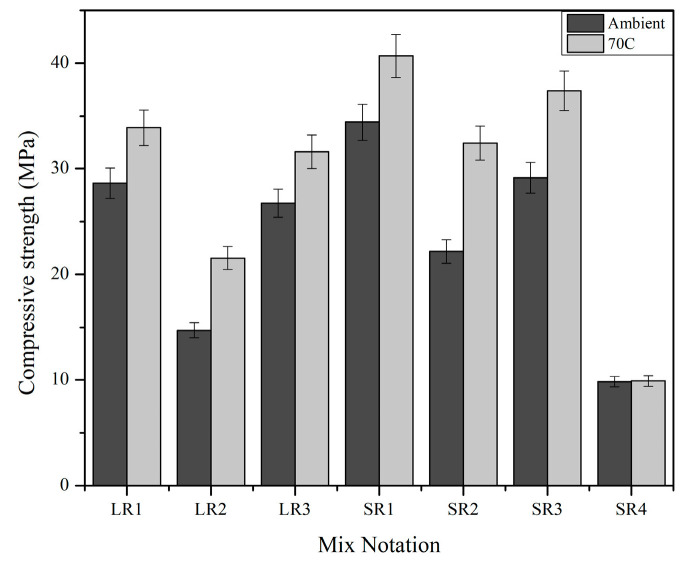
Trend of compressive strength of samples prior to high-temperature exposure.

The compressive strength of the geopolymer mixes prior to the high-temperature exposure under two curing temperatures, ambient room temperature, and at 70 °C is presented in Figure 7. The geopolymer samples cured at 70 °C for 24 h demonstrated superior compressive strength compared with samples cured at ambient room temperature. This trend was observed in all the geopolymer mixes used in this study. The dissolution of geopolymer precursors was slow at ambient temperature, the geopolymer gels developed slowly, and the geopolymer samples were still moist and viscous. Because there was so much water and sol phase in the system, the compressive strength was lower. Raising the curing temperature expedited the production of dense microstructures, notably in the early stages of the geopolymerization reaction, and increased the number of precursors (mainly Al and Si) that were dissolved in the amorphous phases, accelerating the geopolymerization reaction [54]. However, in mix SR4—as the process of mixing potassium hydroxide and rice husk silica did not involve heating liquid potassium hydroxide to dissolve rice husk silica or precipitate potassium silicate and instead involved mixing dry ingredients in a ball mill—there was no strength development at 70 °C curing, as potassium silicate was not produced in situ during the grinding.

### 3.3. Thermal Fatigue Resistance of Geopolymers

**Figure 8:**
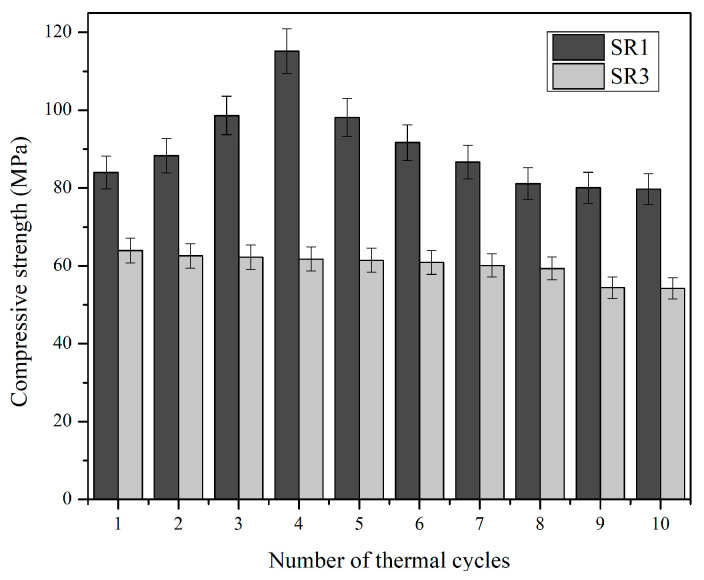
Compressive strength post-thermal cycles.

The compressive strength of the geopolymer samples from mix SR1 was subjected to thermal cycles of ranging from ambient room temperature to 1100 °C. The compressive strength of the samples after each thermal cycle was measured to find the thermal fatigue resistance and durability of the sample upon repeated thermal cycles. Figure 8 shows the values of compressive strength post-thermal-cycle exposure. The value of compressive strength increased with the number of cycles up to the 4^th^ cycle for the geopolymer mix denoted as SR1, following which, a decline in compressive strength and, finally, a stable value at the 9^th^ and 10^th^ thermal cycles were observed, as reported in other thermal fatigue resistance tests [55,56]. The value of compressive strength in the fourth cycle rose to 115.2 Mpa, and the stable compressive strength was around 80 Mpa. The availability of extra SiO_2_ in the chemical composition of mullite facilitated extra geopolymerization reactions and enhanced performance until the fourth cycle in geopolymer mix SR1. Geopolymer mix SR3 had no such trend in strength gain. The value of compressive strength declined gradually in accordance with the thermal cycles until the eighth cycle, after which, the value remained stable at around 54.34 Mpa.

### 3.4. Temperature Distribution Measurement

**Figure 9:**
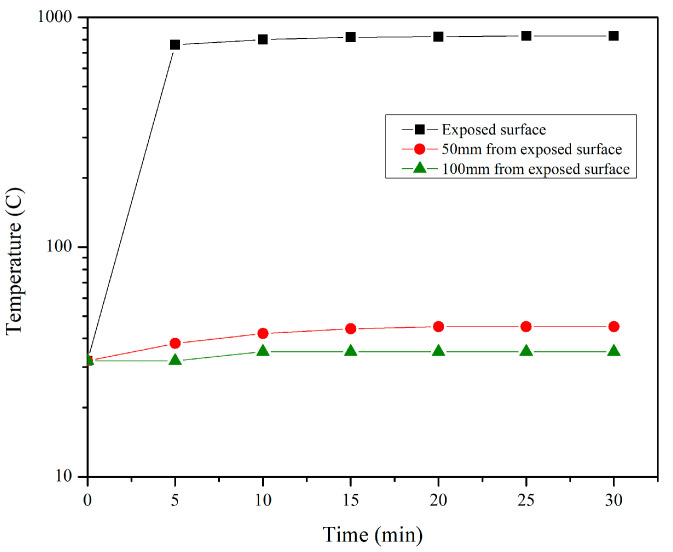
Temperature distribution of SR1 mix under direct flame at 800 °C.

The temperature distribution in the solid geopolymer, upon exposure to a flame at 800 °C, was measured using thermocouples and a heat gun. The measurements were made on a hardened geopolymer sample with a length of 300 mm, a width of 300 mm, and a depth of 150 mm. Two thermocouples at 50 mm and 100 mm from the flame-exposed surface were embedded in the fresh geopolymer sample so it could be exposed to flame after the curing (hardening) period of 7 days. Figure 2b shows a schematic representation of the measurement of temperature distribution in the geopolymer sample. The test was conducted on a hardened geopolymer sample with the mix denoted as SR1 due to its superior mechanical performance post-high-temperature exposure. The distribution of temperature over a period of 30 min is plotted in Figure 9.

In Figure 9, although the exposed surface recorded a rise in temperature of up to 830 °C upon exposure to flame for 30 min, the thermocouple located 100 mm from the exposure face did not record any change in temperature post-exposure for 30 min. The temperature reading of the thermocouple remained at 35 °C, as it was prior to flame exposure. The thermocouple located 50 mm from the exposure face recorded a slight increase in temperature of up to 45 °C in 15 min, and the sample remained stable at that temperature for the rest of the exposure duration, making geopolymer mix SR1 highly heat-insulating in nature. Also, no visual cracks, heat-induced spalling, or concrete projectiles were observed on the exposed surface or anywhere in the sample post-direct flame. 

### 3.5. X-ray Diffraction Results

**Figure 10:**
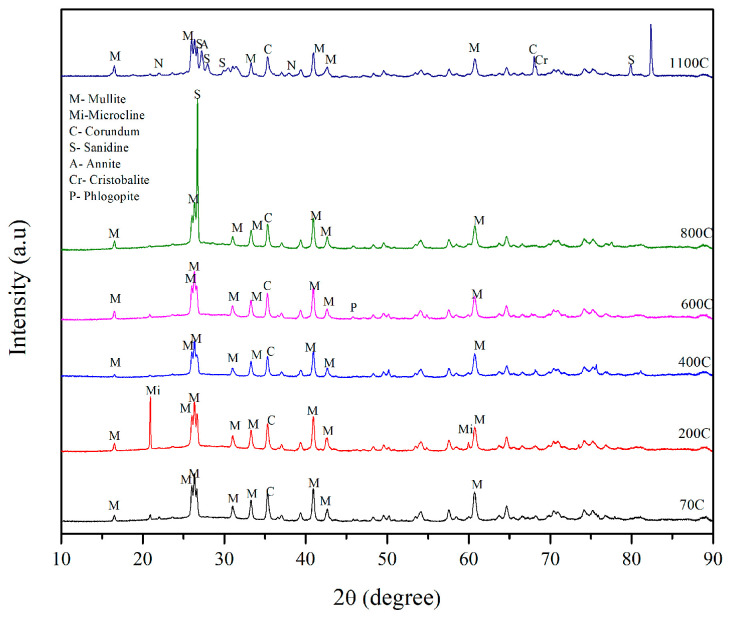
X-ray diffraction plot of SR1 post-temperature exposure.

**Figure 11:**
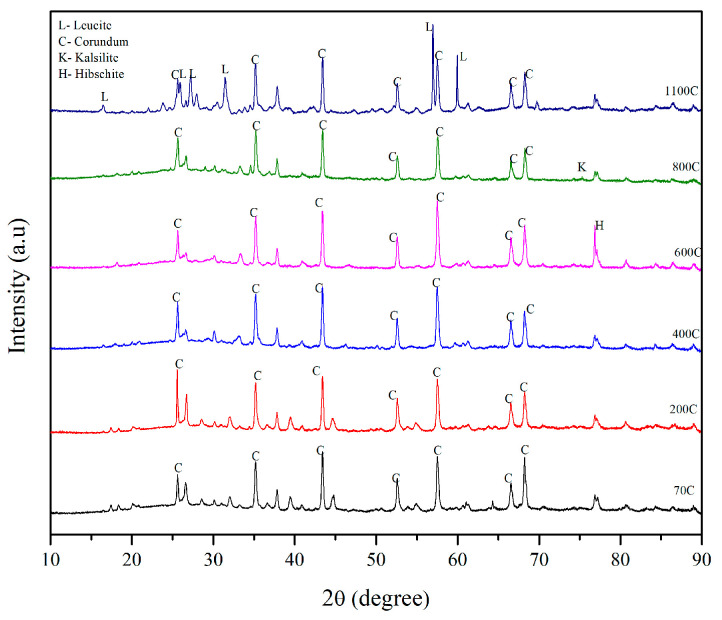
X-ray diffraction plot of SR3 post-temperature exposure.

The results of X-ray diffraction (XRD) studies conducted on mixes SR1 and SR3 after temperature exposures in a furnace are shown in Figure 10 and Figure 11. Figure 10 shows the XRD plot of mix SR1, which had 45-micron mullite as a refractory aggregate in its solid geopolymer matrix. The crystalline phases of corundum and mullite were observed in the sample post-70 °C curing for 24 h and after 6-day ambient curing. At 200 °C, the crystalline phases of microcline were also observed, along with mullite and corundum. At 600 °C, a new phase phlogopite was formed due to viscous sintering, adding some fluidity to the hardened geopolymer sample, thereby reducing its compressive strength, as observed in Figure 6. At 1100 °C, when the strength was highest, the phases of sanidine, annite, and cristobalite were observed in the mix denoted as SR1. Similar phases were reported by Krivenko and Kovalchuk [57] at higher temperatures. These phases have also been mostly observed in metakaolin-based geopolymers exposed to high temperatures [58,59]. 

In Figure 11, an XRD plot of mix SR3 where alumina was used as a refractory aggregate in the solid geopolymer matrix is shown. Most of the crystalline phases in the mix pertained to corundum, except for the formation of hibschite at 600 °C during viscous sintering, Kalsilite at 800 °C, and Leucite at 1100 °C. 

### 3.6. Scanning Electron Microscopy

**Figure 12:**
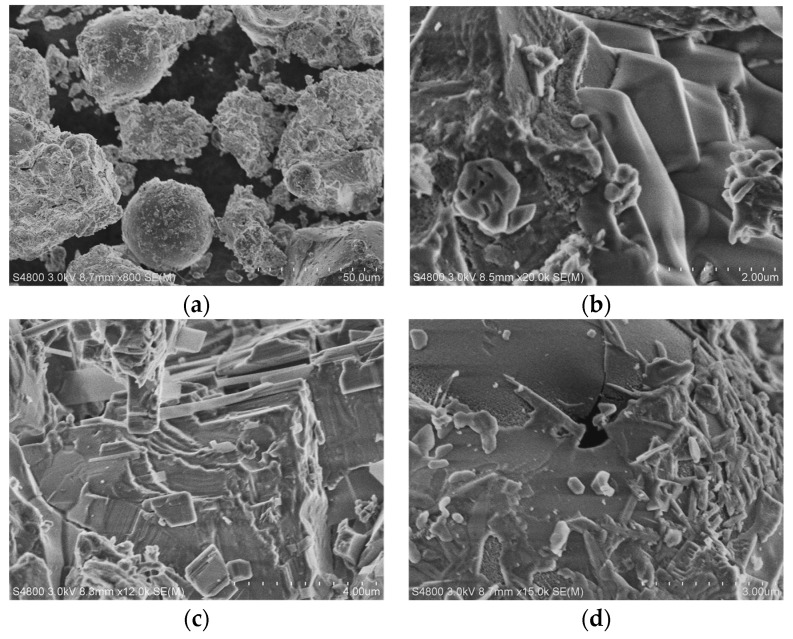
Scanning electron microscopy images of SR1 specimen at 70 °C (**a**), 600 °C (**b**), and 1100 °C (**c**,**d**).

The geopolymer mix denoted as SR1 was chosen to study microstructural changes through scanning electron microscopy imagery. This was performed to identify the phase changes in the geopolymer samples before elevated temperature exposure, during viscous sintering, and after elevated temperature exposure. Figure 12a represents the microstructure of the SR1 specimen before elevated temperature exposure, where spherical fly ash particles can be noticed in the image. Figure 12b shows the formation of thin prismatic laminae, which can be identified as phlogopite, at a temperature of 600 °C. The formation of this phase is an indication of viscous sintering, which reduced the compressive strength of the geopolymer sample. Similar sintering was observed by Hager et al. [46] during their study on applications of high-temperature mortar based on fly ash geopolymers. At 1100 °C, the phases of annite and the recrystallization of mullite into needle-like structures were observed, as can be seen in Figure 12c–d. The recrystallization helped increase the compressive strength by densifying the matrix. Wattanasiriwech et al. [52], in their study, reported similar recrystallization of mullite to needle-like structures in a fly ash-based geopolymer sample.

## 4. Comparative Study of Refractory Potentials of Geopolymers

A short comparison of the refractory potentials of geopolymers in retaining mechanical strength after high-temperature exposure is discussed in this section. Table 4 presents the details of geopolymer mixes—with details on their precursors, activators, and mechanical performance post-high-temperature exposure—for two-part conventional liquid alkaline geopolymers in the literature. Most precursors presented in Table 4 are fly ash, slag, or metakaolin, and only alkaline-activator-based geopolymers are presented to draw comparisons. 

All the above-mentioned literature pertains to two-part liquid alkaline geopolymers where the handling of activators, given their high pH values, can be extremely risky. The design methodology and mixes proposed in the present study negate the risk factor, as the mixes are activated by using water and are easy to handle.

## 5. Conclusions

The present study evaluates advanced solid geopolymer formulations—with fly ash as a precursor, potassium hydroxide and potassium silicate as activators, and mullite and alumina as refractory aggregates—as a potential refractory composite capable of retaining its compressive strength post-high-temperature exposure. Based on the experimental evaluation conducted, the following conclusions have been drawn.

- All the geopolymer formulations used in this study retained their cubical shapes without cracks, spalling, or any physical disintegration post-exposure to 1100 °C for 1 h. The weight loss in the solid geopolymer formulations was more pronounced compared with their two-part liquid alkaline geopolymer counterparts.
- Advanced solid geopolymer formulations yielded better compressive strength after 1100 °C exposure compared with conventional liquid alkaline geopolymer formulations due to intensive mechano-chemical grinding in the process of making advanced solid geopolymers.
- The geopolymer mix made of 45-micron mullite, denoted as SR1, displayed its highest compressive strength, 84 MPa, after 1100 °C, and the crystalline phases of sanidine, annite, and cristobalite were identified in the sample. At 1100 °C, mullite recrystallized as a needle-like structure, densifying the matrix and increasing the compressive strength.
- The solid geopolymer mix made of alumina, denoted as SR3, had leucite as a crystalline phase at 1100 °C, which was responsible for its compressive strength of 64 MPa.
- Geopolymer mix SR1 retained its compressive strength after ten cycles of 1100 °C exposure. Mix SR1 displayed its highest compressive strength, 115.2 MPa, after four cycles, and SR3 had a gradual decrease in strength after each cycle, which further stabilized after eight cycles at 54.4 MPa.
- The temperature distribution profile of mix SR1 proves the superior thermal conductivity of the mix during direct flame exposure.

## Figures and Tables

**Table 1:** Chemical composition and physical characteristics of fly ash.

Oxide Composition (%)					Physical Characteristics		
CaO	SiO_2_	Al_2_O_3_	Fe_2_O_3_	MgO	LOI	Specific Gravity	Strength Activity
2.58	60.70	23.71	4.73	1.14	0.49	2.27	80.77%

**Table 2:** Chemical composition of aggregates.

Aggregate	Al_2_O_3_	SiO_2_	Fe_2_O_3_	CaO	TiO_2_
Mullite	57.41%	40.62%	0.56%	-	1.22%
Alumina	>93%	-	<0.3%	<5.5%	-

**Table 3:** Design mixture proportions in percentage weight (%).

Mix Notation	Fly Ash	Mullite	Alumina	KOH	K_2_SiO_3_	RHS	Water
LR1	26	52	-	6	16	-	-
LR2	26	52	-	6	16	-	-
LR3	27	-	54	5	13	-	-
SR1	26	52	-	3	8	-	11
SR2	26	52	-	3	8	-	11
SR3	27	-	54	2.5	6.5		10
SR4	25	51	-	7	-	4	13

**Table 4:** Refractory potentials of geopolymers as reported by various researchers.

Author	Geopolymer Mix Details	Mechanical Performance
Bezerra and Luz [60]	Geopolymers based on partially or fully replaced calcium aluminate cement (CAC) in high-alumina castables with sodium silicate as an activator.	A dosage of 2.7% weight CAC and 1.3% weight geopolymer at 1400 °C attained a flexural strength of 39.1 MPa.
Farias et al. [61]	Metakaolin-based geopolymers with semi-insulating fused silica-containing castables.	The geopolymer-bonded castable exhibited a flexural strength of 6.24 MPa after exposure to 815 °C.
Deutou et al. [62]	Metakaolin-, calcined bauxite-, and calcined talc-based geopolymer with kyanites of various particle sizes as fillers. Potassium hydroxide and potassium silicate as activators.	An 80 µm kyanite-filler-based geopolymer achieved a flexural strength of 45 MPa at a temperature of 1200 °C.
Ahmed and Kishar [63]	Metakaolin geopolymer pastes incorporated with cement kiln dust and sodium hydroxide and sodium silicate as activators.	Geopolymer mix with 20% cement kiln dust withstood high temperatures with a strength of around 35 MPa at ambient temperature and around 22.5 MPa at 800 °C.
Boum et al. [64]	Metakaolin–bauxite-blended geopolymer with sodium hydroxide and sodium silicate as activators.	The mechanical strength of the samples decreased from 35.2 to 11.1 MPa at room temperature. Compressive strength of 98 MPa at 1200 °C was achieved for a mix with 20% bauxite by weight.
Yaşın and Ahlatcı [65]	Metakaolin-based geopolymer binder reinforced with fine alumina powder, sodium hydroxide, and sodium silicate.	The compressive strength of the sample after 1250 °C exposure was reported to be 134 MPa, whereas the unexposed sample had 30.41 MPa strength.
Moosavi et al. [66]	Metakaolin-based geopolymer with microsilica (25% vol) and tabular alumina aggregates (75% vol). Potassium hydroxide as activator.	Flexural strength was reduced by 23.43 MPa after exposure to 1200 °C. Similar trends were observed in compressive strength with around 40% reduction in strength.
Lahoti et al. [67]	Fly ash geopolymers with sodium and potassium-based activators individually and in combination.	A 30–40% increase in strength in the potassium-activator-based geopolymer, whereas the sodium-activator-based geopolymer reduced in strength (10%) after high-temperature exposure. After exposure to 500 °C, the compressive strength increased from 40 MPa to 59 MPa for the potassium-based geopolymer, and at 900 °C, it reduced to 54 MPa.
King et al. [68]	Metakaolin geopolymer activated by combinations of sodium–potassium silicate and sodium–potassium hydroxide.	Lesser strength losses due to elevated temperature exposures were observed in geopolymers with high Si/Al ratios (>1.5) when they were exposed to 800 °C. The 3-day compressive strength reduction was 4–6%.
Lahoti et al. [69]	Metakaolin-based geopolymers activated by sodium hydroxide and sodium silicate.	Compressive strength drastically decreased at 900 °C. At 25 °C, it was around 65 MPa, and at high temperatures, the strength dwindled to 6 MPa for a mix with a Si/Al ratio of 1.75.
Kong et al. [14]	Metakaolin- and fly ash-based geopolymers with sodium silicate and potassium hydroxide activators.	Fly ash geopolymers increased in strength after 800 °C exposure, whereas metakaolin geopolymers decreased in strength. The metakaolin geopolymer’s unexposed strength was 38.5 MPa, and after exposure, it was 25.4 MPa. Fly ash’s unexposed strength was 59 MPa, and after exposure, it was 62.8 MPa.
Guerrieri and Sanjayan [70]	Fly-ash–slag-based geopolymer in varying dosages with sodium activators.	Geopolymer mix with a 65%/35% (FA/Slag) ratio achieved the highest compressive strength. The residual compressive strength after 800 °C was 20 MPa.
Rickard et al. [71]	Fly ash geopolymers with sodium silicate and sodium aluminate activators.	The compressive strength of samples increased after 1000 °C exposure, wherein the amount of Si or Al added by the activating solution was reduced. The effect was more pronounced in the sodium-aluminate-activated samples, which exhibited strength gains of almost five times, wherein 40% of the total Al was added via activating solution.
Rickard et al. [72]	Fly ash geopolymers with sodium silicate and sodium aluminate activators.	After exposure to 1000 °C, the geopolymer sample exhibited an increase in compressive strength. The unexposed sample had a compressive strength of 33 MPa, whereas, after exposure, the strength increased to 132 MPa.
Rickard et al. [73]	Fly ash geopolymers with sodium silicate and sodium hydroxide activators.	Geopolymers made from unreacted low-strength and low-density fly ash attained better strengths after high-temperature exposure (1000 °C) compared with geopolymer samples made from highly reactive and high-strength fly ash. In the former case, the strength increased from 28 MPa to 93 MPa.

## Data Availability

The data presented in this study are available upon request from the corresponding authors.
